# Learning process of ultrasound-guided Ilio-fascial compartment block on a simulator: a feasibility study

**DOI:** 10.1186/s12245-020-00317-6

**Published:** 2020-11-30

**Authors:** Julien Celi, Christophe A. Fehlmann, Olivier T. Rutschmann, Iris Pelieu-Lamps, Roxane Fournier, Mathieu Nendaz, François Sarasin, Frédéric Rouyer

**Affiliations:** 1 Emergency Unit, Department of Acute Medicine, Geneva University Hospitals, Rue Gabrielle-Perret-Gentil 2, CH-1205 Geneva, Switzerland; 2 Anesthesiology Unit, Department of Acute Medicine, Geneva University Hospitals, Geneva, Switzerland; 3 Unit of Development and Research in Medical Education, Faculty of Medicine, and Department of Medicine, Geneva University Hospitals, Geneva, Switzerland

**Keywords:** Ultrasound-guided fascia iliaca bock, Learning process, Emergency physician

## Abstract

**Background:**

Ultrasound-guided fascia iliaca compartment block (US-FICB) is not part of the learning curriculum of the emergency physicians (EP) and is usually performed by anesthesiologists. However, several studies promote EP to use this procedure.

The goal of this study was to assess the feasibility of a training concept for non-anesthesiologists for the US-FICB on a simulator based on a validating learning path.

**Method:**

This was a feasibility study. Emergency physicians and medical students received a 1-day training with a learning phase (theoretical and practical skills), followed by an assessment phase.

The primary outcome at the assessment phase was the number of attempts before successfully completing the procedure. The secondary outcomes were the success rate at first attempt, the length of procedure (LOP), and the stability of the probe, corresponding to the visualization of the needle tip (and its tracking) throughout the procedure, evaluated on a Likert scale.

**Results:**

A total of 25 participants were included. The median number of attempts was 2.0 for emergency physicians and 2.5 for medical students, and this difference was not significant (*p* = 0.140). Seven participants (28%) succeeded at the first attempt of the procedure; the difference between emergency physicians and medical students was not significant (37% versus 21%; *p* = 0.409). The average LOP was 19.7 min with a significant difference between emergency physicians and medical students (*p* = 0.001). There was no significant difference regarding the stability of the probe between the two groups.

**Conclusion:**

Our 1-day training for non-anesthesiologists with or without previous skills in ultrasound seems to be feasible for learning the US-FICB procedure on a simulator.

## Introduction

Hip fracture is a common pathology in Emergency Departments (ED) and most often involves polymedicated elderly patients with frequent severe co-morbidities [1], https://www.obsan.admin.ch/fr/indicateurs/taux-dhospitalisation-pour-fracture-de-la-hanche.

Despite the fact that effective pain relief is a major challenge in this process, patients are at high risk for not receiving adequate analgesia in a crowded ED [2, 3], especially if they suffer from cognitive disorders [4, 5].

In response to suboptimal pain management that results in oligoanalgesia, various strategies have been developed to improve this situation, some beginning at the ED door, e.g., implementation of a medico-delegated analgesia protocol, campaigns on pain management, fast-track management, and strategies for shortening the time to intervention. Opiate treatment is frequent in the acute phase. Therapeutic equilibrium is not obvious to achieve, which can result in risk of under dosage for fear, secondary effects (delirium, fall, urinary globe), and potential respiratory repercussions. Regional analgesia is a widely recognized analgesia technique for pain analgesia. FICB is a compartmental block allowing excellent analgesia of the proximal part of the femur [6]. Historically performed “blindly” using anatomical landmarks, US-FICB has become a standard [7, 8]. This technique, which is usually performed by anesthesiologists, can be performed by emergency physicians, nurses, and paramedics with relatively little experience [9, 10]. However, there is currently no structured and evidence-based training program for this procedure.

The aim of this project was to assess the feasibility and the effect of a training concept for non-anesthesiologists with or without prior ultrasound skills in the acquisition of technical skills for ultrasound-guided regional anesthesia (UGRA) using a high-fidelity simulator to learn US-FICB.

## Method and materials

### Design and setting

This was a monocentric pilot study in the ED of a primary and tertiary urban teaching hospital in Geneva, Switzerland, that admits 73,000 patients per year.

### Study population

Participants were enrolled on a voluntary basis. Inclusion criteria depended on the profile of the participants. Enrolled emergency physicians had a full position in an emergency division, at least 5 years of postgraduate training, and daily experience in point-of-care ultrasound (group with previous experience in US but not in US-guided regional anesthesia). Enrolled medical students were in their third year of a 6-year medical school program and had not taken, except theoretical lessons, a practical ultrasound course or used an ultrasound machine before (group without previous experience in US).

### Materials

**Fig. 1:**
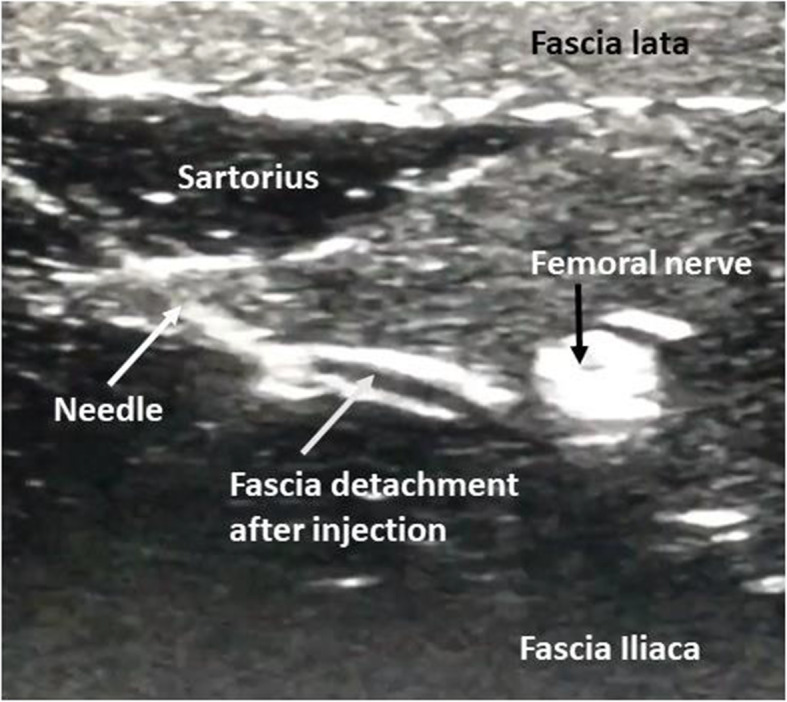
Sono-anatomical landmarks for the simulator/target injection

The high-fidelity simulator used in this study was a Nysora MS2-FEM. This simulator allows hydro dissection and injection under ultrasound control, increasing the level of realism with a high degree of fidelity (Fig. 1). The real-life FICB procedure usually consists of administering 30–40 ml of local anesthetic divided into two syringes. Due to limitations in volume injection on the simulator, it was decided that, for the study, 2 × 5 ml of saline serum would be injected to simulate the handling and changing of syringes. Syringes used were Stimuplex® Ultra 360® 22 Ga (B. Braun Melsungen AG). Linear array ultrasound probes (L12-4 for Philips-SPARQ® and HFL38x for SonoSite, Inc.) were used.

### Teaching session

Four sessions were organized, each with six to eight participants. Each session was under the direct supervision of two instructors, either two attending emergency physicians or one attending emergency physician and one anesthesiologist physician.

**Fig. 2:**
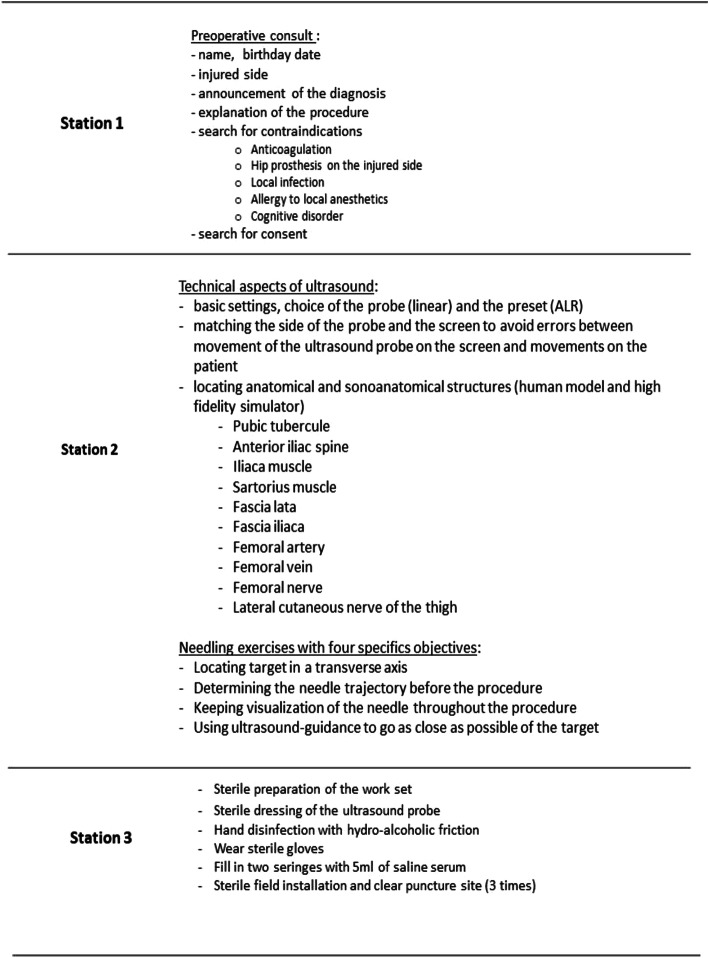
Formative station checklist

A station checklist (Fig. 2) was consensually established by the expert group, which included two senior anesthesiologists, who are experts in UGRA (RF, IP), and two senior emergency physicians (FR, JC).

The teaching session consisted of three parts: one 1-h theoretical session, three 1-h supervised practical sessions, and the viewing of an institutional video showing “the perfect procedure: US-FICB on a high-fidelity simulator.” The participant-to-instructor ratio was 3:1 or 4:1 for practical sessions.

### Assessment session

In the afternoon after training, each participant underwent an individual, timed evaluation on the simulator the participant used previously. Except for one session with only one expert (JC), all the individual assessment sessions were under the supervision of two experts (the same as in the teaching session). JC was present for all sessions. The assessment checklist included 5 steps with a total of 16 control items (Table 1) and was developed by the same expert group that developed the station checklist.

**Table 1:** Assessment checklist

Boxes	Steps	Acquired	Not acquired
***1.*** **Preoperative consult**	Knowledge and compliance for contraindications		
	Explanation of the procedure and obtaining consent		
***2*. Technical aspects**	Choice of ultrasound probe		
	Correct preset selection (loco-regional anesthesia)		
***3*. Asepsis**	Physician’s clothing		
	Preparation of syringes (2 × 5 ml saline serum)		
	Preparation of the probe		
	Correct cleaning of the site		
	Sterile gel for the puncture area		
***4.*** **Ultrasound guidance and anatomical identification**	Correct orientation of the probe		
	*Anatomical and sono-anatomical landmarks		
	*Ideal trajectory and in-plane progression		
	Stability of the probe throughout the procedure* *− 2 = low stability/+ 2 high stability	− 2, − 1, 0, + 1, + 2	
***5.*** **Hydro-dissection procedure**	Visualization of the needle tip throughout the procedure		
	Suction before injection		
	*Identification of the injection area, injection of 2 × 5 cc (syringe change), and visualization of fascia detachment		
**Ultrasound-guided FICB**			
**Number of breakthroughs**			
**LOP**			

Participants had to perform steps 1–3 chronologically to continue the procedure. In case of error, the experts could interrupt the participant to provide verbal feedback, after which the participant had to repeat all the items of the steps that had been performed in error.

The points noted with an asterisk in Table 1 are those identified as critical as they can cause the needle to exit the skin to create a new puncture. Stability of the probe, the only item for which feedback was not given during the evaluation, was evaluated separately by the two experts at the end of the procedure. After comparing their own assessment, which was scored on a 5-level Likert scale from − 2 (low stability) to + 2 (high stability), a consensus was easily found.

Participants had to complete all the items to pass the evaluation (sufficient US-FICB skill acquired). They could not ask the instructor questions except for technical issues. Number of skin breakthroughs was recorded by one of the reviewers. No physical feedback (taking control of the needle) was given.

### Outcomes measure

The primary outcome at the assessment phase after the training was the number of attempts before successfully completing the procedure. An attempt was defined as a skin breakthrough. Success was defined as the completion of every point of the checklist (Table 1) in less than 45 min. Secondary outcomes were successful at first attempt, length of procedure (LOP), and stability of the probe. LOP was defined as the time needed to accomplish the 16 prespecified items listed in Table 2. The stability of the probe was evaluated by the two observers and scored on a scale from − 2 (low stability) to + 2 (high stability). Based on the maximum acceptable duration of a procedure, a procedure longer than 45 min was considered as failed.

**Table 2:** Participant characteristics

Level of expertise	Medical students (*n* = 14)	Emergency physicians (*n* = 11)
Age^a^, years	24 ± 1	34 ± 2
Females, number (%)	5 (36)	4 (36)
Postgraduate experience^a^, year	NA	7 ± 2

^a^Means ± SD

### Statistical analysis

Mann-Whitney-Wilcoxon test and Fischer’s exact test were used for comparisons between groups. For all tests, a two-sided *P* value less than 0.05 was considered significant. Statistical analyses were performed using STATA version 14 (Stata Corporation, TX, USA).

## Results

Twenty-five participants were included: 11 senior emergency physicians and 14 medical students, 64% of whom were male. Participant characteristics are presented in Table 2. All participants were right handed and finished the procedure.

**Fig. 3:**
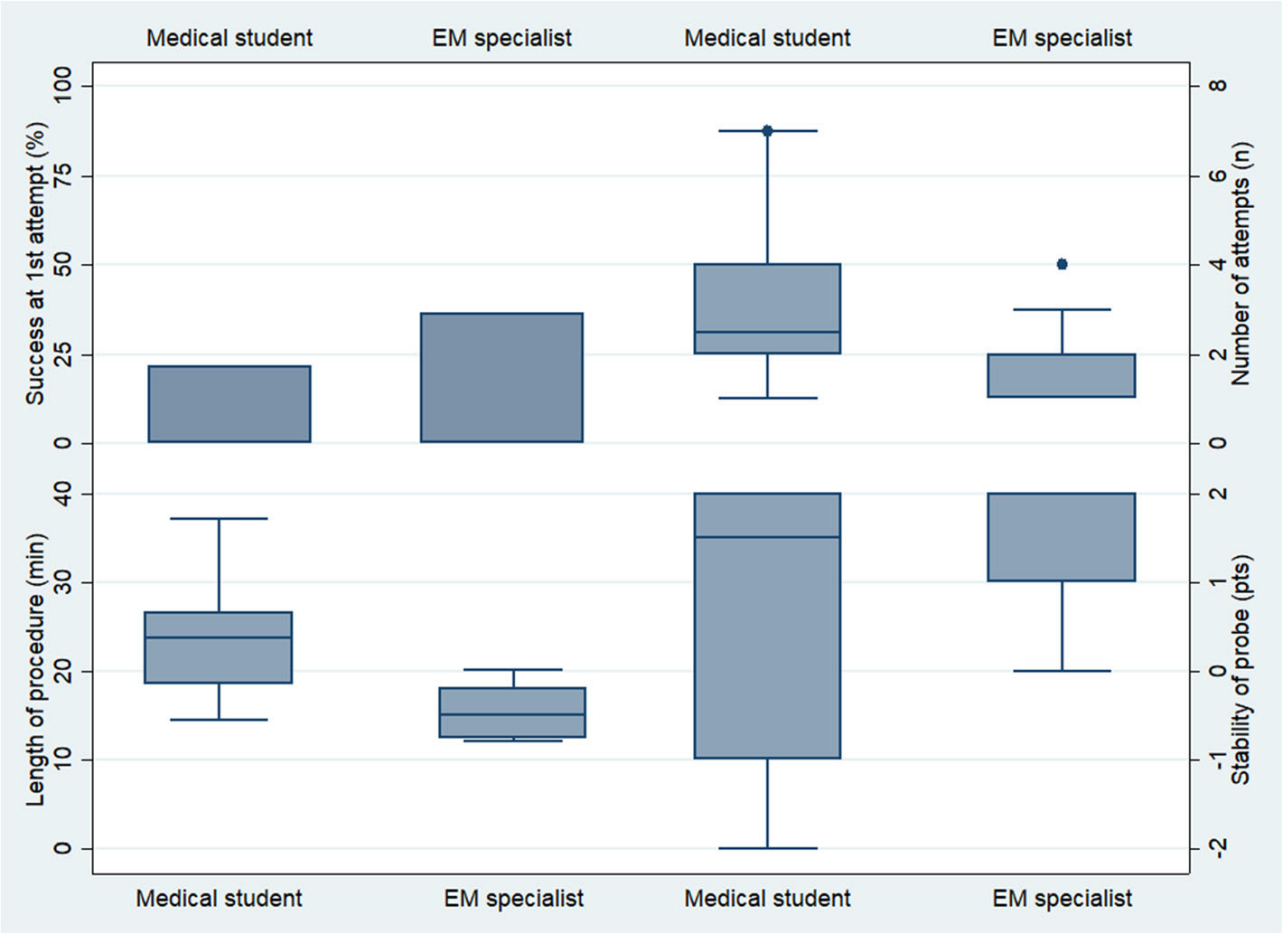
Primary and secondary outcomes at the end of training

Outcome results are shown in Fig. 3 and Table 3.

**Table 3:** Primary and secondary outcomes

Level of expertise	Medical students (*n* = 14)	Emergency physicians (*n* = 11)	*p* value
Number of attempts^a^	2.5 [2.0–3.0]	2.0 [1.0–2.9]	0.140
Success at first attempt, number (%)	3 (21)	4 (37)	0.409
Length of procedure^b^, min	23.2 ± 6.2	15.3 ± 2.9	0.001
Stability of probe^b^, points	0.5 ± 1.7	1.5 ± 0.8	0.215

^a^Median [95% CI]

^b^Means ± SD

For the primary outcome, the median number of attempts was 2.0 (95% CI 1.0–2.9) for emergency physicians and 2.5 (95% CI 2.0–3.0) for medical students, and the difference between the groups was not statistically significant (*p* = 0.140).

For the secondary outcome, the first attempts of seven participants (28%) were successful, and there was a clinically significant difference between emergency physicians and medical students (37% versus 21%; *p* = 0.409).

The average LOP was 19.7 min, with a significantly shorter LOP for emergency physicians (15.3 min, 95% CI 13.4–17.2, versus 23.2 min, 95% CI 19.6–26.8, *p* = 0.001).

Finally, the overall average stability of the probe was 0.9, with no significant difference between emergency physicians and medical students (1.5 versus 0.5, *p* = 0.215).

No vascular or nervous puncture was performed.

## Discussion

This study demonstrates that a 1-day structured, competence-based training seems sufficient for learning US-FICB with a simulator regardless of previous experience in US.

The use of locoregional anesthesia in acute pain management in the ED, particularly of FICB for fractures of the proximal femur, is well-established [11]. The FICB practice is effective and safe and can be performed by non-anesthesiologist [12, 13]. However, there are obstacles to transfer of competence, including the lack of training for emergency doctors in the performance of UGRA. However, this study indicates that providing sufficient training can easily be accomplished.

The observed difference in first attempt success rate between experienced practitioners and medical students, although not statistically significant, was 26% in favor of the experienced group. This is an important difference and the lack of statistical significance likely reflects that the study was seriously underpowered with respect to this outcome (see limitations). This difference can be explained by the previous experience of senior physician in US-guided procedures such as central line. As demonstrated in the study of Kim et al. [14], novice students in UGRA significantly improve their learning curve after five attempts for a simple nerve block (as the FICB) on a simulator; this observation reflects the learning curve of the students in our study (by adding up all the trainings of the day, the individual number of attempts exceeded five per participant). The median number of attempts in this study agrees well with those reported by Morse et al. [15] and Liu et al. [16]. The acquisition of simple technical skills (low difficulty block, as with the FICB) does not seem to require a strong medical background, and this is likely due to the focus in the training on the acquisition of a specific procedural skill.

The stability of the probe was not significantly different between the two groups, even though there was a clear trend that the stability achieved by the students was inferior to that by the physicians. As described by Sites et al. [17], poor stability is among the most frequent errors made by novices in the acquisition of an ultrasound-based gesture in locoregional anesthesia. Note that, during the evaluation session, the instructors provided oral feedback freely on the conduct and monitoring of the probe, particularly to students, and it would have been interesting to measure the impact of such feedback on the performance and reliability of the results obtained. Although it was not addressed in this study, it would be interesting to assess whether the hand-eye coordination obtained by habitual playing of video games influences the learning curve of trainees using the simulator [18].

The LOP required by students was significantly longer than for physicians. This can be explained by the prior experience of the emergency physicians for the preparation of the material, the disinfection of the patient, and the dressing of the probe—experience that the students had not yet gained. Observers had the impression that steps 1–3 (see Table 2) required considerably more time for students to complete. To ensure that the assessment provides realistic data for a competency-based curriculum, a total procedure time of maximum 45 min was chosen for this study.

The training curriculum developed in this study was based on the most recent evidence for technical learning in the medical field, on the contribution of simulation to learning UGRA and on expert recommendations in good practice of the FICB [19–23]. To our knowledge, this study is the first to compare the acquisition of skills by physicians and medical students for US-FICB using a simulator.

Some limitations should, however, be acknowledged. First, the most important limitation is the likely lack of statistical power of the study.

Secondly, the participants in each experience group were unbalanced in number and gender. Emergency physicians generally had a wide range of proficiency in US. With a larger number of participants, future studies should determine the skill level of physicians before training to meaningfully measure the impact of this program.

Thirdly, different co-examiners performed evaluation in the assessment sessions, potentially adding bias to the evaluations. This potential bias was limited as the main author acted as a co-examiner for all evaluations. Despite the fact that various studies have measured the positive impact of combining verbal and physical feedback on performance during skill learning [24], we chose to only give verbal feedback in this study. We did not measure the number of times feedback was given, although such data could be interesting with a larger number of participants. Although the impact of simulation in the transfer of skills learned in clinical practice is clearly appreciated for most teaching models and undeniably provides added value with respect to the perception of the learners, the real impact of simulation is yet to be determined [19, 25]. Another limitation of this study could be the extrapolation of the results to clinical practice, as a single size of manikin was used and is not representative of all the actual sono-anatomical landmarks encountered in clinical practice. In addition, the assessment session took place on the same day as the teaching session, and, therefore, only the short-term effect of the training was evaluated. In future studies, a second assessment session 1 or 3 months later could be added to evaluate long-term effects, including a questionnaire evaluating the impact of the training in the implementation of the technique in the clinical practice of the physicians included in the study as well as their degree of satisfaction. Although the US-FICB is carried out autonomously by EPs in different countries, our EPs do not have the prerequisite to do so. We have therefore not been able to assess how many physicians have included this technique in their clinical practice or even assess the maintenance of competence. However, we hope that our study can be the starting point of the training for non-anesthesiologists in our hospital.

Finally, fatigability of participants, unmeasured in this study, could have influenced performance [18].

## Conclusion

This study demonstrates the feasibility of a training concept for non-anesthesiologists with or without prior US skills for the performance of US-FICB on a high-fidelity simulator. More studies involving real patients and long-term effect are needed to evaluate transfer of competence.

## Data Availability

The data that support the findings of this study are publicly available upon request.
